# Regulation of morphogen pathways by a *Drosophila* chondroitin sulfate proteoglycan Windpipe

**DOI:** 10.1242/jcs.260525

**Published:** 2023-04-11

**Authors:** Woo Seuk Koh, Collin Knudsen, Tomomi Izumikawa, Eriko Nakato, Kristin Grandt, Akiko Kinoshita-Toyoda, Hidenao Toyoda, Hiroshi Nakato

**Affiliations:** ^1^ Department of Genetics, Cell Biology and Development, University of Minnesota, Minneapolis, MN 55455, USA; ^2^ Faculty of Pharmaceutical Sciences, Ritsumeikan University, Shiga 525-8577, Japan

**Keywords:** Chondroitin sulfate proteoglycan, Windpipe, Decapentaplegic, Wingless, Morphogen signaling, *Drosophila*

## Abstract

Morphogens provide quantitative and robust signaling systems to achieve stereotypic patterning and morphogenesis. Heparan sulfate (HS) proteoglycans (HSPGs) are key components of such regulatory feedback networks. In *Drosophila*, HSPGs serve as co-receptors for a number of morphogens, including Hedgehog (Hh), Wingless (Wg), Decapentaplegic (Dpp) and Unpaired (Upd, or Upd1). Recently, Windpipe (Wdp), a chondroitin sulfate (CS) proteoglycan (CSPG), was found to negatively regulate Upd and Hh signaling. However, the roles of Wdp, and CSPGs in general, in morphogen signaling networks are poorly understood. We found that Wdp is a major CSPG with 4-*O*-sulfated CS in *Drosophila*. Overexpression of *wdp* modulates Dpp and Wg signaling, showing that it is a general regulator of HS-dependent pathways. Although *wdp* mutant phenotypes are mild in the presence of morphogen signaling buffering systems, this mutant in the absence of Sulf1 or Dally, molecular hubs of the feedback networks, produces high levels of synthetic lethality and various severe morphological phenotypes. Our study indicates a close functional relationship between HS and CS, and identifies the CSPG Wdp as a novel component in morphogen feedback pathways.

## Teaser

**Summary:** The *Drosophila* chondroitin sulfate proteoglycan Windpipe finetunes heparan sulfate-dependent morphogen signaling, indicating a close functional relationship between chondroitin sulfate and heparan sulfate.

## INTRODUCTION

Heparan sulfate (HS) and chondroitin sulfate (CS) are the most evolutionarily conserved glycosaminoglycans (GAGs) that are found in diverse animal species, including *Caenorhabditis elegans*, *Drosophila* and mammals. HS and CS are long, unbranched polysaccharides, composed of repeating disaccharide units: GlcA–GlcNAc and GlcA–GalNAc, respectively. They exist as forms of proteoglycans (PGs) in which one or more GAG chains are covalently attached to specific serine residues on the core protein. Both types of PGs are found on the cell surface and in the extracellular matrix.

It has been well established that heparan sulfate proteoglycans (HSPGs) function as co-receptors for growth factor signaling, and regulating the distribution and reception of secreted signaling proteins (Esko and Selleck, 2002; Kirkpatrick and Selleck, 2007; Li and Kusche-Gullberg, 2016; Lindahl and Li, 2009; Xu and Esko, 2014). The list of ‘HS-dependent factors’, secreted ligands that require HSPG co-receptors for proper distribution and signaling, continues to grow. Interestingly, many of these factors function as morphogens: a special type of signaling molecules that direct different cell fates in a concentration-dependent manner. *In vivo* studies using the *Drosophila* model have shown that HSPGs regulate gradient formation and signaling of four key morphogen molecules: Decapentaplegic (Dpp; a *Drosophila* BMP), Wingless (Wg; a *Drosophila* Wnt), Hedgehog (Hh) and Unpaired (Upd, or Upd1; a ligand of the Jak/Stat pathway) (Nakato and Li, 2016). During development and homeostasis, these same molecules also function as ‘niche factors’ that control stem cell self-renewal and differentiation in the stem cell niches. Therefore, HSPG co-receptors play critical roles in orchestrating various stem cell behaviors (Bowden and Nakato, 2021). For example, Dally, a *Drosophila* HSPG of the glypican family, serves as a Dpp co-receptor and regulates Dpp gradient formation in the developing wing (Akiyama et al., 2008; Belenkaya et al., 2004; Fujise et al., 2003) as well as the female germline stem cell niche (Guo and Wang, 2009; Hayashi et al., 2009). Interestingly, as *dally* expression is repressed by Dpp signaling, Dally forms a negative feedback loop of this pathway (Fujise et al., 2003). Similarly, the expression of *thickveins* (*tkv*), encoding a Dpp receptor, is regulated by morphogen signaling itself (Lecuit and Cohen, 1998). Such multiple circuits of feedback loops are believed to contribute to the robustness of the morphogen systems (Eldar et al., 2003; Irons et al., 2010; Lander et al., 2007; Lei and Song, 2010; Nakato and Li, 2016). Expression of *dally* is also controlled by Wg and Hh signaling, two additional pathways that Dally regulates (Fujise et al., 2001). Thus, Dally acts as a molecular hub of morphogen feedback networks.

HSPG function is tightly regulated by its biosynthetic and post-biosynthetic modification events. In the Golgi apparatus, a series of modification steps add sulfate groups to specific ring positions of HS. The degree and patterns of sulfation have a major impact on the activity of HS (Xu and Esko, 2014). In addition to these reactions in the Golgi apparatus, HS structure is further modified on the cell surface by the extracellular endosulfatases, Sulfs, in a post-biosynthetic manner (Ai et al., 2003; Dhoot et al., 2001; Kamimura et al., 2006; Uchimura et al., 2006). Sulfs specifically remove sulfate groups at the 6-*O* position of glucosamine residues from highly sulfated regions of HS. In *Drosophila*, a single Sulf homolog, Sulf1, modulates FGF, Wg, Hh and Egfr signaling during development (Butchar et al., 2012; Dani et al., 2012; Kamimura et al., 2006; Kleinschmit et al., 2010, 2013; Takemura and Nakato, 2017; Wojcinski et al., 2011; You et al., 2011). In the developing wing, Sulf1 negatively regulates Wg signaling by removing ligand binding sites on HS (Kleinschmit et al., 2010). Importantly, expression of *Sulf1* is induced by the Wg pathway itself. A similar phenomenon has been also reported for the Hh and Vein-Egfr pathways (Butchar et al., 2012; Wojcinski et al., 2011). Thus, like Dally, Sulf1 is another molecular hub of morphogen feedback circuits.

Compared to HS, much less is known regarding the role of CS in cell signaling (Cortes et al., 2009; Townley and Bülow, 2018). In *Drosophila*, only a few molecules have been shown to bear CS chains, which include Kon-tiki (Kon) (Losada-Perez et al., 2016; Perez-Moreno et al., 2014, 2017), Multiplexin (Mp) (Csordas et al., 2020; Harpaz et al., 2013; Momota et al., 2011) and Windpipe (Wdp) (Ren et al., 2015; Takemura et al., 2020). Given the structural similarities between CS and HS, chondroitin sulfate proteoglycans (CSPGs) might have modulatory, supportive and/or complementary functions to HSPGs. In fact, Wdp, a single-pass transmembrane CSPG with leucine-rich repeat motifs, modulates Upd and Hh signaling (Ren et al., 2015; Takemura et al., 2020). This raised the idea of a ‘dual PG co-receptor system’ in which HS-dependent pathways are also generally regulated by CS (Coles et al., 2011; Takemura et al., 2020). Interestingly, *wdp* expression is induced by Upd-Jak/Stat signaling in the midgut, forming a negative feedback loop in this pathway (Ren et al., 2015). This suggests a possibility that CSPGs might function together with HSPGs as morphogen feedback regulators. However, the functional relationship between HSPGs and CSPGs remains elusive.

In the current study, to gain insights into the function of CSPGs in morphogen signaling and their relationship with HSPG co-receptors, we performed biochemical and genetic analyses of Wdp. We found that Wdp is a major CSPG in *Drosophila*, which bears 4-*O*-sulfated CS chains and regulates HS-dependent pathways. When we perturbed morphogen feedback loops by introducing a *Sulf1* or *dally* mutation, *wdp* mutation resulted in severe morphological defects, including abnormal patterning of wing blade and hinge structures, egg retention in the ovary and a wing posture abnormality. Our results implicate Wdp as a novel player of the morphogen feedback buffering systems.

## RESULTS

### Two types of CSPGs in *Drosophila*

**Fig. 1.**
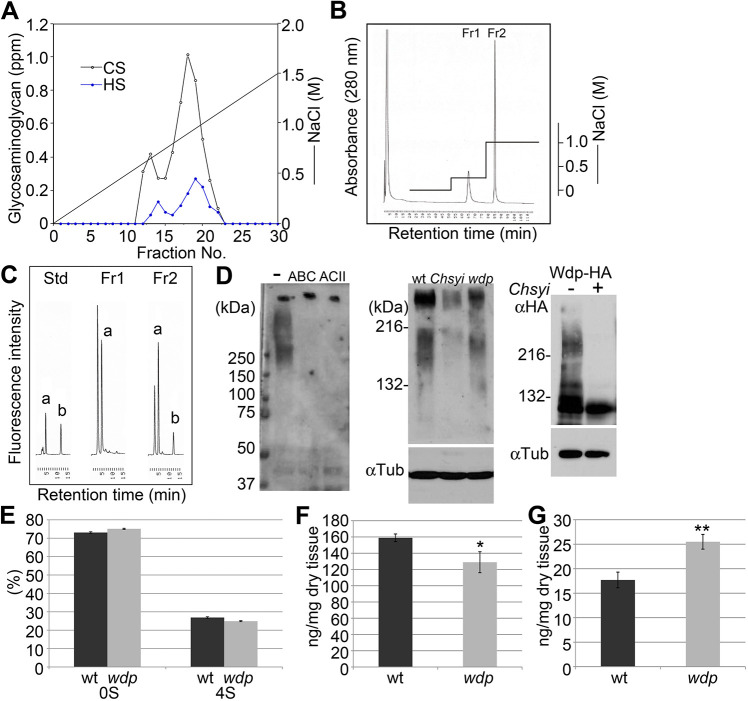
**Wdp is a major 4-*O*-sulfated CSPG in *Drosophila*.** (A) Separation of glycosaminoglycans in adult *Drosophila* on a DEAE column. The crude GAGs were prepared from adult flies and applied to a HiPrep DEAE column. Bound GAG fractions were eluted with a 0–1.5 M NaCl gradient. (B) Anion-exchange chromatography of *Drosophila* CSPGs. CSPGs were isolated from adult flies and applied to a HiTrap TM DEAE FF column. Bound PG fractions were eluted stepwise with 0.26 M and 1.0 M NaCl to obtain fractions 1 (Fr1) and 2 (Fr2), respectively. The elution patterns are shown by absorbance at 280 nm. (C) Chromatograms of unsaturated disaccharides from *Drosophila* CS. CS was prepared from fractions 1 (Fr1) and 2 (Fr2), and completely digested with chondroitinase ABC. The resultant disaccharide species were separated by reversed-phase ion-pair chromatography (Docosil C22) with post-column detection system and compared to the standard (Std). The following disaccharides are shown: ΔDi-0S (a) and ΔDi-4S (b). (D) Immunoblot analysis of *Drosophila* CSPGs. Left panel: protein extracts from wild-type (wt) adult flies were subjected to immunoblot analysis using an anti-CS antibody (LY111) (wild type). The same sample was examined before (−) and after the treatment with chondroitinase ABC or ACII. Middle panel: protein extracts from *actin>Chsy* RNAi (*Chsyi*) and *wdp* mutant (*wdp*) animals were also analyzed. Anti-α-tubulin antibody was used for an internal control. Right panel: Wdp protein from Wdp–HA (*Chsy* RNAi−) and Wdp–HA, *actin>Chsy* RNAi (*Chsy* RNAi+) was detected using an anti-HA antibody. (E–G) Disaccharide analyses of *wdp* mutant GAGs. CS (E,F) and HS (G) were purified from wild-type (wt; black bars) and *wdp* mutant (*wdp*; gray bars) adult flies and disaccharide species were quantified. CS disaccharide composition (0S, ΔDi-0S; 4S, ΔDi-4S) (E) and total amounts of CS (F) and HS (G) (both ng/mg dry tissue) are shown. The amount of CS and HS in wt and wdp flies were analysed in triplicate. *P<0.05; **P<0.01 (Welch's two-sided, unpaired *t*-test).

As the first step of biochemical analyses of *Drosophila* GAGs, crude HS and CS samples were prepared from adult flies and analyzed by anion exchange chromatography. The GAGs bound to a DEAE column were eluted with a 0–1.5 M NaCl gradient. The elution patterns showed two peaks both in HS and CS samples (Fig. 1A), suggesting that *Drosophila* GAGs can be largely separated into two groups with different charges. A similar pattern was also observed in the analysis of CSPGs. DEAE-column chromatography of CSPGs purified from adult flies using a stepwise elution with different salt concentrations (0.26 and 1.0 M NaCl) clearly separated the CSPG specimen into two fractions, fractions 1 and 2 (Fig. 1B).

To characterize the two fractions of *Drosophila* CSPGs, we analyzed the CS structures by disaccharide analysis. Briefly, CS was purified from fractions 1 and 2 as shown in Fig. 1B, and completely digested into disaccharides by chondroitinase ABC. The resultant disaccharide species were separated and quantified by reversed-phase ion-pair chromatography with a post-column detection system (Dejima et al., 2013b; Kamimura et al., 2006; Kleinschmit et al., 2010; Nakato et al., 2019; Toyoda et al., 2000). In *Drosophila*, two major CS disaccharide species, unsulfated (ΔDi-0S) and 4-*O*-sulfated (ΔDi-4S) disaccharide units, can be detected but 6-*O* sulfation of GalNAc residues is under the detection limit (Toyoda et al., 2000). We found that CS isolated from fraction 2 contains ΔDi-4S, but this disaccharide was not detectable in fraction 1 CSPGs (Fig. 1C). This observation indicates that there exists two groups of CSPGs in *Drosophila*: one bearing 4-*O*-sulfated CS and another with non-sulfated chondroitin. This is a unique feature in *Drosophila* CSPGs.

### Wdp is a major 4-*O*-sulfated CSPG

We found that a commercially available anti-CS antibody (LY111) detects *Drosophila* CS. This is one of few antibodies that can recognize *Drosophila* GAGs and offers a useful tool to study the biological functions of CS using this model organism. Immunoblot analysis of whole-protein extracts from wild-type adults using LY111 detects high-molecular-mass proteins (>200 kDa) as smear bands (Fig. 1D, left panel). These smear bands disappeared after the treatment of samples with chondroitinase ABC or chondroitinase ACII (Fig. 1A), confirming that the smear bands represent CSPGs.

We also performed an RNAi knockdown for *Chsy*, a *Drosophila* homologue of human ChSy-1, a key component of CS polymerases (Kitagawa et al., 2001; Mikami and Kitagawa, 2013; Sugahara et al., 2003) (Fig. 1D, middle panel). A *UAS-Chsy RNAi* transgene was driven using a ubiquitous *actin-Gal4* driver. The LY111 signal was substantially reduced in *Chsy* knockdown animals (*act>Chsy RNAi*). Interestingly, the extract prepared from *wdp* mutants showed a significantly decreased amount of CS compared to wild type. In general, loss of a single PG core protein does not reduce GAG-positive bands from the whole animals or organs as detected bands represent the sum of sugar moieties of a large number of PG molecules. This result suggested that Wdp might be one of the major CSPGs in *Drosophila*. In fact, high-throughput expression analyses in FlyBase (see http://flybase.org/cgi-bin/rnaseqmapper.pl?dataset=celniker_wiggle&xfield1=FBgn0034718 and http://flybase.org/cgi-bin/rnaseqmapper.pl?dataset=tissues_stranded&xfield1=FB gn0034718) indicate that this gene is expressed at very high levels in many tissues and developmental stages.

Wdp was identified as a CSPG in our previous study via a glycoproteomic approach (Takemura et al., 2020). To confirm that Wdp is modified with CS by immunoblot analysis, we used a transgenic strain, *wdp-HA* (previously called *wdp^KI.HA^*) (Takemura et al., 2020). In this strain, HA-epitope-tagged Wdp protein is expressed from its endogenous locus. Anti-HA antibody staining of ovary protein extract detected smear bands (Fig. 1D, right panel, wdp-HA). When we blocked CS biosynthesis by *Chsy* RNAi knockdown in *wdp-HA* animals (*wdp-HA*, *act>Chsy RNAi*), the smear bands were lost. Instead, a single band representing the Wdp core protein was detected. This result confirmed a CS modification of Wdp.

To determine the amount of CS and its disaccharide composition in *wdp* mutants, we performed disaccharide analyses. We found that the disaccharide composition of CS from *wdp* mutants was comparable to that of wild type (Fig. 1E; Table S2). However, the total amount of CS was reduced by approximately 19% in *wdp* mutants (Fig. 1F). The reduction of CS in *wdp* mutants is consistent with our western blot results and supports the idea that Wdp is a major CSPG in this animal.

Similar analyses of HS showed that HS disaccharide composition is unchanged in *wdp* mutants (Table S3). Interestingly, however, the total amount of HS was increased by approximately 44% in the mutant (Fig. 1G; Table S3). In many model systems, genetic manipulations that reduce HS result in increased production of CS (Bachvarova et al., 2020; Bai et al., 1999; Holmborn et al., 2012; Le Jan et al., 2012; Lidholt et al., 1992; Lin et al., 2000). Therefore, it is possible that the reduction of CS is compensated by the elevated synthesis of HS.

### Localization of 4-*O*-sulfated CS in the developing wing

**Fig. 2.**
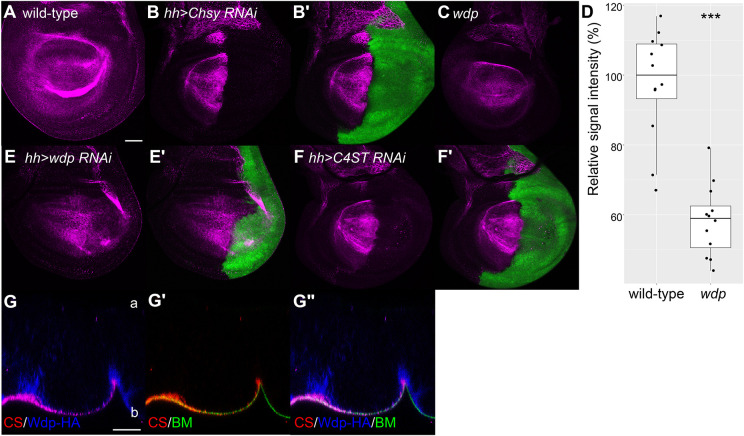
**Localization of 4-*O*-sulfated CS in the developing wing.** (A–C) Wing discs from wild type (A), *hh>Chsy RNAi* (B,B′) and *wdp* mutant (C) were stained with the anti-CS antibody (LY111, magenta). *UAS-GFP* expression marks the *hh-Gal4*-expressing posterior compartment (green, B′). (D) Quantification of the LY111 signal in wild-type and *wdp* mutant wing discs. Relative intensity for the anti-CS staining signal was compared between wild-type and *wdp* mutant wing discs. The signal was calculated from a central region of the wing pouch in each genotype and measured using ImageJ (*n*=12 wing discs for each genotype). Boxes indicate the 25–75th percentiles, and the median is marked with a line. The whiskers extend to the highest and lowest values within 1.5 times the interquartile range. (E–F′) Wing discs from *hh>wdp RNAi* (E,E′) and *hh>C4ST RNAi* (B,B′) were stained with anti-CS (LY111, magenta). *hh-Gal4*-expressing cells are marked by GFP (E′,F′). (G–G″) Apicobasal distribution of CS was examined by confocal *z*-section imaging of wing disc cells from a larva bearing *wdp-HA* and *trol-GFP*, a BM marker. Anti-CS (LY111), anti-HA and GFP signals are shown in red, blue and green, respectively. Positions of apical (a) and basal (b) membranes are marked. Scale bars: 50 μm (A); 20 μm (G). Images are representative of 10–20 wing discs. ***P<0.001 (Welch's two-sided, unpaired *t*-test).

To analyze spatial distribution of CS in a tissue, we stained the wing discs using LY111. In wild type, the LY111 signal was detected uniformly throughout the wing disc (Fig. 2A). To ask whether the LY111 signal indeed reflects the distribution of CS, we blocked CS biosynthesis specifically in the posterior compartment of the wing disc. Expression of *UAS-Chsy RNAi* was driven by *hh-Gal4*, a posterior compartment-specific driver. We observed that RNAi knockdown of *Chsy* eliminated the LY111 signal (Fig. 2B,B′), confirming the specificity of LY111 staining in immunohistochemistry.

We next analyzed *wdp* mutant wing discs with the LY111 antibody. We observed a significant reduction in the LY111 signal intensity in the mutant discs compared to that in wild type (Fig. 2C). Quantification of the signal intensity confirmed that, consistent with our immunoblot analysis, the LY111 signal was significantly decreased in *wdp* mutants (Fig. 2D). This reduction was also confirmed by RNAi knockdown. Expression of *UAS-wdp RNAi* in the posterior compartment partially but significantly decreased the signal intensity (Fig. 2E,E′).

The anti-CS antibody (LY111) is believed to recognize highly sulfated structures of CS, such as CS-A units or 4-*O*-sulfated CS [GlcUAβ1–3GalNAc(4S)] (Deepa et al., 2007). Therefore, we asked whether the LY111 signal might be affected by blocking CS sulfation. *CG31743* is orthologous to several human genes, including *CHST11*, and is predicted to encode a *Drosophila* homologue of CS 4-*O* sulfotransferase (C4ST). We found that RNAi knockdown of *CG31743* abolished the epitope of this antibody, similarly to *Chsy* RNAi (Fig. 2F,F′). This result supports that LY111 detects a 4-*O*-sulfated CS *in situ* and confirms that *CG31743* encodes a *Drosophila* C4ST. Hereafter, the gene *CG31743* is referred to as *C4ST*.

It is worth noting that the LY111 signal was detected in a cell-autonomous manner in both RNAi knockdown treatments; GFP signals from gene-specific RNAi-expressing cells showed no overlap with LY111 signals (Fig. 2B,E,F). This is important information because it implies that major CSPGs in this tissue are either integral membrane PGs or secreted PGs that are embedded in the ECM in proximity to the expressing cells.

We next analyzed the distribution of CS in further detail, along the apicobasal axis of the wing epithelium. Our previous study has shown that Wdp is enriched in the basal membranes of epithelia, including wing cells (Takemura et al., 2020). As shown in Fig. 2, the LY111 signal largely overlaps with endogenously expressed epitope-tagged Wdp (Wdp–HA) (Fig. 2G,G″). In addition, the LY111 signal is also detected in the basement membrane (BM) layer, visualized by a protein trap line of Perlecan (*trol-GFP*) (Fig. 2G′,G″) (Medioni and Noselli, 2005). No significant signal was observed at the apical side of the epithelium in this organ. Thus, this observation showed that CSPGs are mainly localized in the basal membrane and the BM of the developing wing.

### Role of CS 4-*O* sulfation on Wdp function

**Fig. 3.**
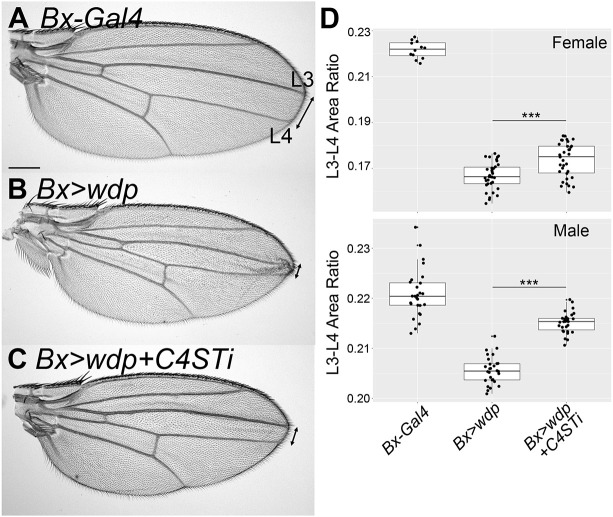
**Role of CS 4-*O* sulfation on Wdp function.** Adult wings of *Bx-Gal4* (A), *Bx>wdp* (B) and *Bx>wdp, C4ST RNAi* (C) are shown. Longitudinal wing veins L3 and L4 are shown. The area enclosed by L3 and L4 veins was calculated and divided by the whole wing area in each wing. This value, the L3-L4 area ratio, was quantified in each genotype (D). The top and bottom graphs show the results for female and male wings, respectively (*n*=12–32 wings for females, *n*=27 wings for each genotype for males). Boxes indicate the 25–75th percentiles and the median is marked with a line. The whiskers extend to the highest and lowest values within 1.5 times the interquartile range. Scale bar: 200 μm. ***P<0.001 (Welch's two-sided, unpaired *t*-test).

Our biochemical data as well as immunohistochemical observations suggest that CS chains of Wdp are 4-*O* sulfated. To determine the contribution of CS 4-*O* sulfation to Wdp function, we used RNAi knockdown of *C4ST*. Our previous study showed that *wdp* overexpression using *Bx-Gal4* impairs Hh signaling, resulting in reduced central area of the wing between longitudinal wing veins L3 and L4 (Takemura et al., 2020) (Fig. 3A,B). Using this assay system, we addressed whether co-expression of *UAS-C4ST RNAi* with *UAS-wdp* affects Wdp activity as a negative regulator of Hh signaling.

We found that *C4ST* RNAi knockdown showed a statistically significant impairment of Wdp activity in inhibiting Hh signaling (Fig. 3C,D), demonstrating the importance of CS 4-*O* sulfation for the full activity of Wdp. Interestingly, *C4ST* RNAi did not completely rescue the L3–L4 area, interfering with the Wdp activity only partially. This might suggest a possibility that CS chains with no 4-*O* sulfation retain residual activity to modulate Hh signaling. However, although this *UAS-C4ST RNAi* construct efficiently reduced the LY111 signal in the wing disc (Fig. 2F), it is always possible that a partial effect is due to, at least partly, incomplete efficacy of RNAi. A future study with a *C4ST* null mutation will clarify this point.

### Effects of *wdp* overexpression on Dpp signaling

Wdp was previously shown to regulate Jak/Stat and Hh signaling, two HS-dependent pathways (Ren et al., 2015; Takemura et al., 2020). These observations raised the question of whether this CSPG is a general regulator of morphogen pathways, also affecting signaling events of other HS-dependent factors, such as Dpp and Wg.

**Fig. 4.**
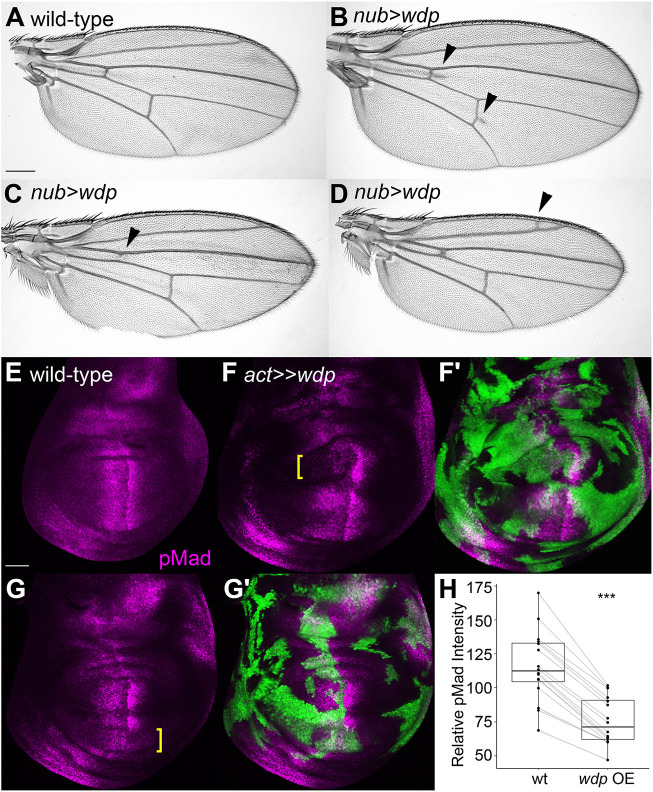
**Effect of Wdp overexpression on Dpp signaling.** (A–D) Adult wings of wild type (A) and *nub>wdp* (B-D) are shown. Arrowheads mark the positions of wing vein defects. (E–G′) Wild-type wing disc (E) and two examples of wing discs bearing *wdp*-expressing flp-out clones (F–G′) stained with anti-pMad antibody (magenta). The flp-out clones are marked with *UAS-GFP* expression (green in F′,G′). Examples of the areas in which *wdp* overexpression downregulated pMad levels are indicated by yellow brackets. (H) Boxplots showing the effect of *wdp* overexpression clones on pMad signal intensity. pMad staining signal intensity in randomly selected *wdp* overexpression (OE) clones was compared with that in immediate neighboring wild-type cells along the anterior–posterior axis (*n*=16 pairs). Boxes indicate the 25–75th percentiles and the median is marked with a line. The whiskers extend to the highest and lowest values within 1.5 times the interquartile range. Scale bars: 200 μm (A); 50 μm (E). ***P<0.001 (Welch's two-sided, unpaired *t*-test).

When we used *nubbin (nub)-Gal4* to drive *UAS-wdp* expression, we observed a few different phenotypes we did not see in *Bx>wdp*. One class of the new phenotypes was wing vein defects, including a spur of ectopic venation at the anterior and posterior crossveins (Fig. 4A,B), loss or reduction of crossveins (Fig. 4C), and the formation of extra vein materials, most frequently near the longitudinal wing vein 2 (L2) (Fig. 4D). These wing vein phenotypes are characteristic of Dpp signaling defects and commonly observed in HS-related mutants (Dejima et al., 2013b; Takeo et al., 2005).

In our previous study, we attempted to determine the role of Wdp in Dpp or Wg signaling by overexpressing *UAS-wdp* in the developing wing using *apterous-Gal4* or *hh-Gal4* (Takemura et al., 2020). However, as *wdp* overexpression in a large region of a tissue from an early developmental stage changed the shape of the tissue, this analysis was inconclusive. Therefore, we employed the flp-out technique to generate *wdp*-overexpressing clones in random locations of the wing disc (Bowden et al., 2022; Struhl and Basler, 1993). We stained these wing discs with an antibody specific to the phosphorylated form of the Mad protein (anti-pMad antibody), which serves as a direct readout of Dpp signaling (Fig. 4E–G′). We found that within the *wdp*-overexpressing clones induced in the central region of the wing discs where cells receive high levels of Dpp signaling, pMad levels were reduced compared to those in neighboring regions outside the clones (yellow brackets in Fig. 4F,G, quantification in Fig. 4H). This result indicated that Wdp downregulates Dpp signaling.

### Effects of *wdp* overexpression on Wg signaling

**Fig. 5.**
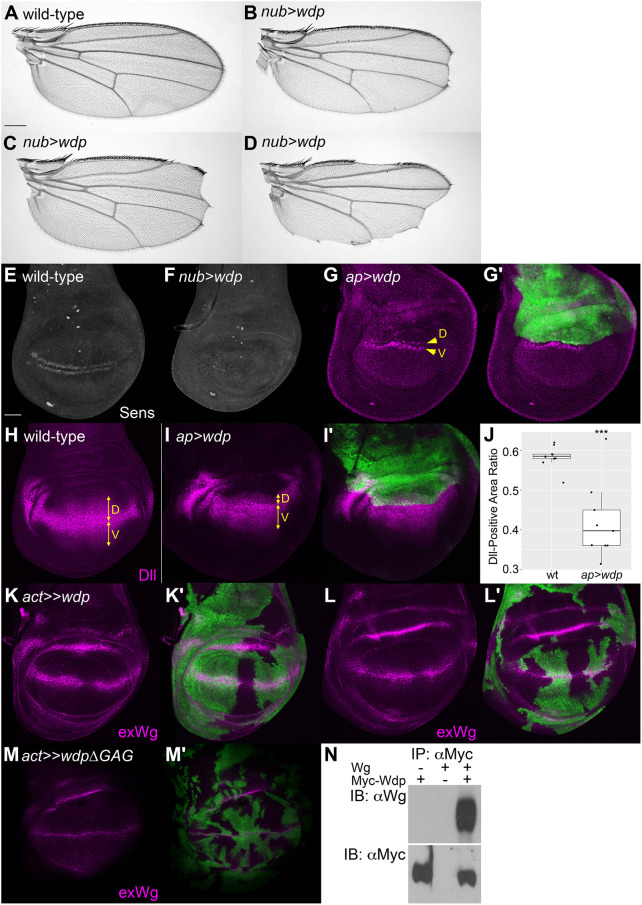
**Effect of Wdp overexpression on Wg signaling.** (A–D) Adult wings of wild type (A) and *nub>wdp* (B–D) are shown. (E–G′) Wild-type (E), *nub>wdp* (F) and *ap>wdp* (G,G′) wing discs were stained with anti-Sens antibody. The rows of Sens-positive cells in the dorsal (‘D’) and ventral (‘V’) compartments are marked in G. (H–I′) Wild-type (H) and *ap>wdp* (I,I′) wing discs were stained with anti-Dll antibody. The positions of the dorsal and ventral compartments are marked by yellow lines. (J) Quantification of the ratio of the Dll-expressing domain in the dorsal wing pouch cells in wild-type and *ap>wdp* wing discs (*n*=9 wing discs for wt and *ap>wdp*). Boxes indicate the 25–75th percentiles and the median is marked with a line. The whiskers extend to the highest and lowest values within 1.5 times the interquartile range. (K–L′) Two examples of wing discs bearing *wdp*-expressing flp-out clones stained with anti-Wg antibody (magenta) using an extracellular staining protocol. (M,M′) Example of a wing disc bearing *WdpΔGAG*-expressing flp-out clones stained with anti-Wg using an extracellular staining protocol. The flp-out clones are marked with *UAS-GFP* expression (green in G′,I′,K′–M′). (N) Complex formation of Wdp and Wg. Wg was expressed in S2 cells with or without a secreted form of Myc–Wdp. Proteins were recovered from conditioned medium using an anti-Myc antibody and precipitates were blotted and probed with anti-Wg antibody. Wg was recovered in the precipitate when co-expressed with Myc–Wdp. Images are representative of 10–20 wings (A–D) or 10–20 wing discs (E–I, K–M′). IB, immunoblotting; IP, immunoprecipitation. Scale bars: 200 μm (A); 50 μm (E). ***P<0.001 (Welch's two-sided, unpaired *t*-test).

In addition to the vein phenotypes, *wdp* overexpression using *nub-Gal4* resulted in wing notching, or a deletion of a part of the wing margin structure, indicative of the impairment of Wg signaling (Fig. 5A–D). To determine whether Wdp is involved in Wg signaling *in vivo*, we examined expression of Senseless (Sens), a high-threshold target of Wg signaling, in the *wdp*-overexpressing wing discs. In wild type, the Sens protein is expressed in two stripes of cells near the dorsoventral boundary of the wing disc (Fig. 5E). We found that Sens expression at the dorsoventral border was severely impaired by *nub-Gal4*-mediated overexpression of *wdp* (Fig. 5F). Sens expression in other regions, which is not dependent on Wg signaling, was not affected by Wdp. When *wdp* was overexpressed specifically in the dorsal compartment using the *ap-Gal4* driver (*ap>wdp*), anti-Sens staining was diminished only in the dorsal row (‘D’), leaving the ventral row (‘V’) intact (Fig. 5G,G′, yellow arrowheads). This was also confirmed by staining *ap>wdp* discs with anti-Distal-less (Dll), a low-threshold target of Wg signaling. In wild type, Dll-positive cells were distributed evenly in the dorsal and ventral compartments (Fig. 5H). In *ap>wdp* discs, Dll expression was severely diminished in the dorsal compartment (Fig. 5I,J). Taken together, consistent with the adult wing phenotypes, these results showed that Wdp downregulates Wg signaling.

In a mammalian tissue model, Wnt-3a is known to bind to a highly sulfated structure of CS and 4-*O* sulfation, which affects Wnt-3a diffusion (Nadanaka et al., 2008, 2011). To determine whether Wdp, which bears 4-*O*-sulfated CS chains, affects the distribution of the Wg ligand, we stained wing discs bearing *wdp* flp-out clones with the anti-Wg antibody using the extracellular staining protocol (Kleinschmit et al., 2010; Strigini and Cohen, 2000). This protocol specifically visualizes the Wg ligand in the extracellular space. Surprisingly, we found a significant increase in the level of extracellular Wg protein within *wdp* flp-out clones (Fig. 5K–L′). Thus, *wdp* overexpression increases Wg ligand levels while downregulating its signaling.

To determine whether this function of Wdp requires CS chains, we generated flp-out clones overexpressing WdpΔGAG. In the *wdp*Δ*GAG* construct, all serine residues required for CS attachment were substituted with alanine residues so that the core protein is not modified with CS (Takemura et al., 2020). We found that WdpΔGAG failed to increase the level of extracellular Wg protein (Fig. 5M,M′). These observations strongly suggest that Wdp overexpression sequesters the Wg ligand via CS chains and reduces the pool of ligand molecules available to form the functional ligand/receptor/co-receptor signaling complex, consistent with the idea that HS and CS competitively function to finetune Wg signaling.

We next examined whether Wdp interacts with Wg *in vitro* by coimmunoprecipitation experiments. We generated a construct for a secreted form (the extracellular domain) of Myc–Wdp (sec-Myc–Wdp) by deleting the transmembrane domain and the intracellular domain. Wg was expressed with or without sec-Myc–Wdp in S2 cells. We found that Wg was immunoprecipitated from conditioned medium with the anti-Myc antibody only in the presence of sec-Myc–Wdp (Fig. 5N). This result indicates that Wdp forms a complex with Wg, further supporting the idea that Wdp sequesters Wg. Taken together, these results showed that Wdp is a general regulator of morphogen signaling pathways that are known to be HS dependent.

### *wdp* null mutation causes wing patterning defects in the absence of *Sulf1*

Although *wdp* overexpression disrupts Dpp and Wg signaling, *wdp* null mutants do not show obvious defects on these pathways. It is well known that morphogen pathways are controlled by multiple circuits of feedback regulation to buffer against genetic and environmental perturbations, and thus are highly robust (Eldar et al., 2003; Fujise et al., 2003; Kleinschmit et al., 2010; Lander et al., 2007). We hypothesized that the loss of *wdp* is compensated by modulation of HS-related genes, masking *wdp* mutant phenotypes. To test this idea, we examined the genetic interactions between *wdp* and HS-related genes to determine what happens if this buffering system is compromised by breaking this feedback loop. We chose two HS-related genes for this analysis, *Sulf1* and *dally*, as the protein products of both genes were previously shown to be molecular hubs extensively involved in morphogen feedback networks (Butchar et al., 2012; Fujise et al., 2003; Kleinschmit et al., 2010; Wojcinski et al., 2011; You et al., 2011).

**Fig. 6.**
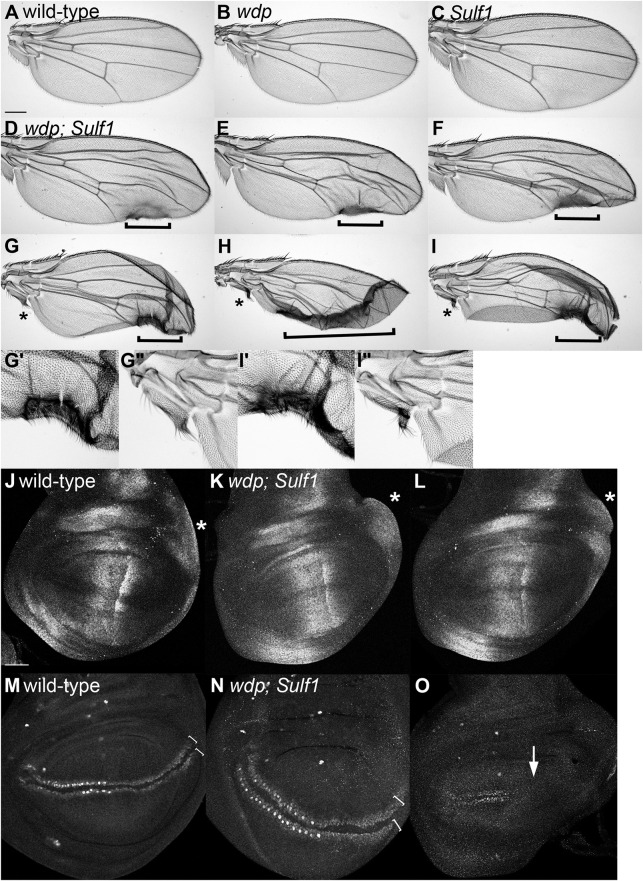
***wdp; Sulf1* double mutants show wing defects.** (A–I″) Adult wings of wild type (A), *wdp* (B) and *Sulf1* (C), and six examples of wings from *wdp; Sulf1* double mutants (D–I″) are shown. Two specific regions of the wing affected in the double mutants, the posterior wing margin (D–I) and alula (in G–I), are marked by brackets and asterisks, respectively. These wing regions in G,I are shown with high-magnification views in G′,G″ and I′,I″. (J–O) Third instar wing discs from wild type (J,M) and *wdp; Sulf1* double mutant (K,L,N,O) were stained with anti-pMad (J–L) and anti-Sens (M–O) antibodies. Asterisks indicate the region of pMad-positive cells induced by posterior Dpp (J–L). Brackets show the range of Sens-positive cells at the dorsoventral border in the posterior compartment (M,N). The arrow shows the lack of Sens-positive cells (O). Images are representative of 10–20 wings (A–I) or 10–20 wing discs (J–O). Scale bars: 200 μm (A); 50 μm (J).

Among the *Drosophila* HS-modifying enzymes, Sulf1 is known to inhibit most, if not all, HS-dependent pathways, including Wg, Hh, BMP and Upd-Jak/Stat signaling, by removing the ligand binding sites on HS (Kleinschmit et al., 2010; Wojcinski et al., 2011). We first realized that *wdp; Sulf1* double mutants were highly lethal. Although both *wdp* or *Sulf1* single mutants were homozygous viable and fertile, the lethality of the double mutant was higher than 95%. In addition, adult survivors showed various morphological defects, including adult wing abnormalities. As reported before (Takemura et al., 2020), *wdp* mutant wings did not show any gross morphological defects (Fig. 6A,B). Similarly, *Sulf1* mutant wings did not show any patterning defects, although the mutant wings were slightly larger than wild-type wings (Fig. 6C) (Dejima et al., 2013a). In contrast, in *wdp; Sulf1* double-mutant survivors, the wing patterning was massively disrupted. Although the severity of the phenotypes varied between individuals, the penetrance of these defects was 100%. Interestingly, we found that two specific regions of the wing were affected. First, the medial–distal part of the posterior edge of the wing was deleted, abnormally pigmented and often had ectopic bristles (Fig. 6D–I, brackets). Second, similar defects, including pigmentation and ectopic bristles, were observed in the alula, a structure at the posterior hinge region (Fig. 6G–I, asterisks).

### Molecular basis for the *wdp; Sulf1* double-mutant wing defects

Given that the Hh pathway regulates patterning in the anterior compartments, these posterior defects of the *wdp; Sulf1* double mutants cannot be explained by altered Hh signaling. The formation of alula is controlled by Dpp derived from the posterior compartment (Foronda et al., 2009). Therefore, we examined Dpp signaling in *wdp; Sulf1* double-mutant wing discs using the anti-pMad antibody. We found that overall levels of pMad staining were higher in the double mutant compared to the wild type (Fig. 6J–L). Asterisks mark the region where cells receive posterior Dpp signaling, which directs alula formation. We observed modest overgrowth in this region of the double-mutant discs.

As wing notching and ectopic bristles are commonly observed with altered Wg signaling, we next asked whether this pathway is affected in the *wdp; Sulf1* double-mutant wing discs. Anti-Sens antibody staining revealed two classes of phenotypes in the mutants (Fig. 6M–O). In the first group, the two rows of Sens-positive cells were more broadly spread in the posterior region of the double-mutant discs (Fig. 6N). The distance between the two rows was also larger. These observations suggest a broader Wg gradient. In the second group, the posterior Sens signals were lost and the disc was severely deformed, suggesting reduced Wg signaling (Fig. 6O). Thus, these discs appeared to show both upregulation and downregulation of Wg signaling. This is not uncommon in HS-related gene mutants in which the shape of a morphogen gradient is altered. In addition, the Wg pathway is known to trigger non-autonomous inhibitory signals (Piddini and Vincent, 2009). Thus, reduced Wg signaling in a cell can increase signaling dosage in the surrounding cells. We propose that altered signaling of both Dpp and Wg contribute to the wing-patterning defects of the *wdp; Sulf1* double mutants.

### Ovulation failure in *wdp; Sulf1* double-mutant females

**Fig. 7.**
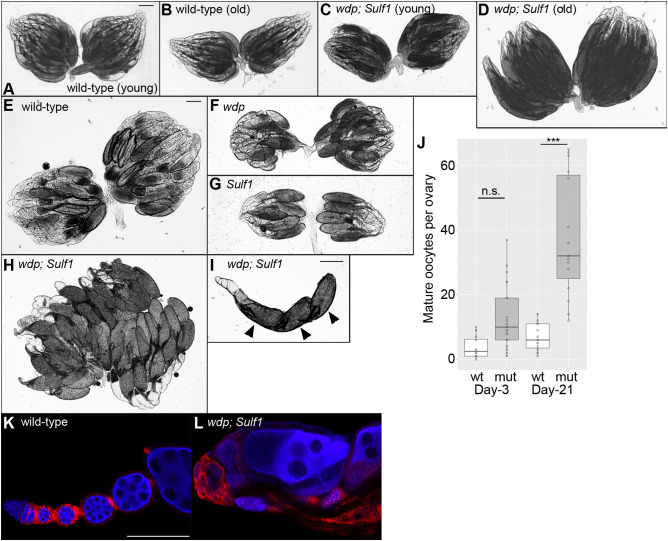
**Ovulation failure in *wdp; Sulf1* double-mutant females.** (A–D) Light microscopy images of an intact pair of ovaries from wild-type (A,B) and *wdp; Sulf1* double-mutant (C,D) females. The ovaries were dissected from young (day 3; A,C) and old (day 21; B,D) animals. (E–H) Light microscopy images of a flattened pair of ovaries from day-21 wild-type (E), *wdp* (F), *Sulf1* (G) and *wdp; Sulf1* double-mutant (H) females. *wdp; Sulf1* animals showed enlarged ovaries, associated with the accumulation of mature oocytes (H). (I) An example of a single ovariole from an old *wdp; Sulf1* double mutant, showing three mature eggs at the posterior end with the dorsal appendages (arrowheads). These ovarioles lacked egg chambers with intermediate stages. (J) The number of mature oocytes were quantified for young (day 3) and old (day 21) wild-type (wt) and *wdp; Sulf1* double-mutant (mut) ovarioles (*n*=8 for wt day 3, 16 for mut day 3, 11 for wt day 21, and 15 for mut day 21). Boxes indicate the 25–75th percentiles and the median is marked with a line. The whiskers extend to the highest and lowest values within 1.5 times the interquartile range. (K,L) Confocal images of ovarioles from day-21 wild-type (K) and *wdp; Sulf1* (L) female flies. Ovarioles were stained with anti-Vas (blue) and anti-Fasciclin 3 (red) antibodies. Images are representative of 10–20 ovaries (A–H) or 10–20 ovarioles (I–L). Scale bars: 200 μm (A,E,I); 100 μm (K). n.s., not significant; ***P<0.001 (Welch's two-sided, unpaired *t*-test).

In addition to a high level of lethality and abnormal wing morphology, *wdp; Sulf1* adult female survivors were completely sterile. We therefore examined ovary morphology of these mutants. Young wild-type females have a pair of ovaries, each of which consists of 16–20 ovarioles, a string of progressively developing egg chambers (Fig. 7A). At the anterior tip of each ovariole is a structure called the germarium that contains the germline stem cells and follicle stem cells. At the posterior edge, ovarioles are connected to the oviduct through which mature eggs are transported to the uterus. During aging, the ovary reduces in size and oogenesis slows (Fig. 7B). We found that the overall morphology of the ovary from young *wdp; Sulf1* double mutants was relatively normal (Fig. 7C). Surprisingly, however, the double-mutant ovaries from aged animals (day 21 after eclosion) were significantly larger compared to wild-type ovaries (Fig. 7D).

We next flattened the ovaries from day-21 females to visualize the composition of the egg chambers (Fig. 7E–H). Under light microscopy, oocytes in the egg chamber of stage 9 (as described in Prasad et al., 2007) and later were visualized as light gray (Fig. 7E). In addition, mature eggs (stage 14) could be recognized by a dark gray color and with fully developed dorsal appendages. We found that ovaries from aged single mutants of *wdp* as well as *Sulf1* were smaller than wild-type ovaries (Fig. 7F,G). In striking contrast, flattened specimens of aged *wdp; Sulf1* double mutants were much larger than those of wild type, with an abnormally higher number of mature eggs (Fig. 7H). Quantification of mature oocytes in an ovariole showed that this ‘egg retention’ phenotype is age dependent (Fig. 7J). Typically, an aged double-mutant ovariole contained three or more mature eggs at the posterior end (Fig. 7I), whereas a wild-type ovariole had one. Although the germarium existed, egg chambers with intermediate stages (stage 7–13 oocytes) were lost (Fig. 7I). In fact, this is a common characteristic of mutants that have egg-laying defects (Liao and Nässel, 2020; Monastirioti, 2003).

We next immunostained mutant ovarioles with anti-Vas (germline cells) and anti-Fasciclin 3 antibodies (follicle cells) and observed them by confocal microscopy. In old wild-type females, ovarioles show normal progression of oogenesis in ordered egg chambers (Fig. 7K). Fig. 7L shows an example of an ovariole from an aged *wdp; Sulf1* double mutant, in which the organization and morphology of the egg chambers were massively disrupted. The lack of intermediate-stage egg chambers and accumulation of mature eggs were also confirmed (data not shown). These observations indicate that simultaneous loss of *wdp* and *Sulf1* results in the failure of ovulation – the transport of mature eggs from the ovary to the oviduct – leading to the swollen-ovary phenotype.

### Genetic interactions between *wdp* and *dally*

**Fig. 8.**
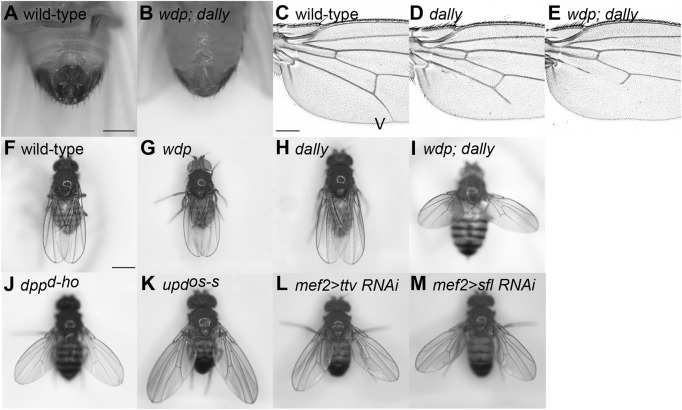
**Outstretched wing phenotype of *wdp; dally* double mutant.** (A,B) Ventral views of adult male abdomen for wild-type (A) and *wdp; dally* (B). *wdp; dally* double mutants showed complete loss of male external genitalia. (C–E) Longitudinal wing vein V is shown for wild-type (C), *dally* (D) and *wdp; dally* (E). (F–M) Resting wing posture is shown for wild-type (F), *wdp* (G), *dally* (H), *wdp; dally* (I), *dpp^d-ho^* (J), *upd^os-s^* (K), *mef2>ttv RNAi* (L) and *mef2>sfl RNAi* (M) flies. *wdp; dally* double mutants showed an ‘outstretched wing’ phenotype, whereas *wdp* and *dally* single mutants showed normal wing posture. Images are representative of 10–20 flies (A,B, F–M) or 10–20 wings (C–E). Scale bars: 250 μm (A); 200 μm (C); 500 μm (F).

We next analyzed the genetic interactions between *wdp* and *dally*, another feedback hub of HS-dependent morphogen pathways. We first found that *wdp* significantly enhanced some, but not all, *dally* mutant phenotypes. For example, 93.5% of males of *wdp; dally* mutants showed a complete lack of external genitalia (Fig. 8A,B), whereas only 3.3% of *dally* mutant males showed this phenotype. Also, the expressivity of a wing vein defect of *dally* mutants was strongly enhanced in the double mutants (Fig. 8C–E). *dally* mutants showed an incomplete longitudinal wing vein V, lacking its most distal portion (Nakato et al., 1995) (Fig. 8D). In *wdp; dally* mutant wings, the deletion of wing vein V extended into the proximal region and it often failed to reach the posterior cross vein (Fig. 8E). Interestingly, however, the penetrance of the wing-notching phenotype of *dally* mutants was not affected by the *wdp* mutation (data not shown).

In addition to the effects on known *dally* phenotypes, we also found that *wdp; dally* double-mutant adults showed a defect in resting wing posture, or the ‘outstretched wing’ phenotype. This phenotype has never been reported in any HS-related gene mutants. In the double mutants, wings were held out from the body at a 45–90° angle from the longitudinal body axis, whereas in the single mutants, the wings were held over the abdomen (Fig. 8F–I). It is worth mentioning that classical mutants showing the same phenotype include *dpp^d-ho^* and *upd^os-s^* (Fig. 8J,K) (St Johnston et al., 1990; Wang et al., 2014). These are hypomorphic alleles of *dpp* and *upd*, respectively, two genes encoding morphogen ligands that use Dally as a co-receptor (Fujise et al., 2003; Hayashi et al., 2012).

This observation indicated that HS-dependent morphogen pathways are required for normal wing posture. Outstretched wing posture can be caused by altered flight muscle function and physiology (Everetts et al., 2021). Therefore, we hypothesized that the morphogen pathways function for flight muscle development. To test this idea, we inhibited HS biosynthesis in the developing muscles by RNAi knockdown of one of two HS biosynthetic genes, *tout-velu* (*ttv*, encoding a HS co-polymerase) and *sulfateless* (*sfl*, encoding a *N*-deacetylase/*N*-sulfotransferase). Expression of either *UAS-ttv* RNAi or *UAS-sfl* RNAi by the *mef2-Gal4* driver recapitulated the outstretched-wing phenotype (Fig. 8L,M). Taken together, our results show that in the absence of *Sulf1* or *dally*, *wdp* mutation led to a high level of lethality and morphological defects that were normally rescued by the feedback buffering system.

## DISCUSSION

Morphogens are a class of signaling molecules that form concentration gradients in a developmental field and specify different cell fates in a concentration-dependent fashion. Many of these pathways can become oncogenic when hyperactivated. Therefore, the signaling dosage of these pathways has to be tightly controlled during development for proper patterning as well as cancer prevention. One of the key features of the morphogen system is its robustness: multiple circuits of feedback regulation buffer against genetic and environmental perturbations. HSPG co-receptors play critical roles in quantitative control of morphogen signaling output as well as feedback control (Nakato and Li, 2016). On the other hand, the functions of CSPGs in morphogen signaling of the genetically tractable model organism *Drosophila* are largely unknown.

Our study showed that *Drosophila* CSPGs are classified into two groups: PGs with or without 4-*O*-sulfated CS. Wdp is a major 4-*O*-sulfated CSPG and regulates Dpp and Wg signaling, two major pathways regulated by HSPGs. We found that *wdp* overexpression increased the extracellular level of the Wg ligand, which resulted in reduced expression of downstream targets of Wg signaling. This finding suggests that Wdp downregulates Wg signaling by sequestering the Wg protein. Thus, in this context, CS appears to compete with HS to control the amount of the ligand available to activate the receptors.

Despite the obvious phenotypes of *wdp*-overexpressing animals, the effect of *wdp* null mutation in Dpp- or Wg-dependent specification events was not evident. However, in the absence of Sulf1 or Dally, two known molecular hubs of morphogen feedback networks, a *wdp* mutation produced synthetic lethality and various morphological and physiological phenotypes. These data indicate that the feedback systems of morphogen-HSPG signaling provide buffering effects and can compensate for the lack of the CSPG Wdp. Thus, Wdp is not only a general regulator of HS-dependent pathways but also a novel component of the morphogen feedback regulatory network. The identification of Wdp as a specific CSPG molecule that regulates all four key HS-dependent pathways suggests that HS-dependent factors might be generally controlled by both HS and CS (Coles et al., 2011; Takemura et al., 2020). CSPGs appear to provide additional layers of morphogen regulation, which is likely to finetune signaling dosage and provide the robustness of cell signaling as well as developmental programs.

As Wdp affects multiple HS-dependent factors, *wdp* mutations offer an interesting opportunity to genetically analyze the HS-CS relationship. We found that double mutations in *wdp* in combination with an HS-related gene exhibited three novel, unique phenotypes that have never been observed in single mutants of genes encoding HSPGs and HS biosynthetic enzymes: (1) deletion, pigmentation and ectopic bristle formation in specific regions of the wing; (2) egg retention in the ovary; and (3) an outstretched wing phenotype. There are a few possible mechanisms by which ovulation is impaired in aged *wdp; Sulf1* double mutants. Ovulation is controlled by octopaminergic neural signaling, which activates the contraction of ovary and oviduct muscles to push a mature egg from the posterior end of the ovary into the oviduct (Lim et al., 2014; Monastirioti, 2003; Sun and Spradling, 2013). Therefore, simultaneous loss of *wdp* and *Sulf1* might disrupt a step in this pathway or normal muscle development in these organs. Alternatively, ovulation might be impaired by physical disruption in *wdp; Sulf1*, such as the failure of the formation of a tubular structure that connects the ovary and oviduct (Deady et al., 2015; Kiss et al., 2019). *Drosophila* ovulation and flight muscle development will be additional useful model systems to study the functions of HS and CS.

As both Wdp and Sulf1 have inhibitory activities on HS-dependent pathways (Kleinschmit et al., 2010; Takemura et al., 2020), it was reasonable to observe that they genetically enhanced each other. In this regard, it is interesting that *wdp* enhanced a specific set of *dally* mutant phenotypes. Dally acts as a co-receptor for morphogen ligands to promote signaling. At the same time, however, it limits their diffusion (Fujise et al., 2003). It is possible that *wdp* enhanced *dally* by aggravating the gradient formation and local availability of morphogen ligands.

In vertebrates, CSPGs are well established as major structural components of connective tissues, including cartilage, and support their mechanical cushioning properties. Vertebrate CSPGs are also known to regulate cell signaling by binding to growth factor ligands (Li et al., 2011; Mizumoto et al., 2015; Nadanaka et al., 2008; Whalen et al., 2013) or activating cell surface receptors (Izumikawa et al., 2014; Mikami et al., 2009; Mizumoto et al., 2012, 2015). CS, like HS, is evolutionarily old and shared by more primitive animal species that have no cartilage, bone or skin. It is intriguing to know how CS emerged during evolution and what its original roles were. One possibility is that HS and CS were partners as signaling regulators in an ancestral species. Further studies of CSPGs in various invertebrate model organisms such as *Drosophila* will help provide insight into these questions.

## MATERIALS AND METHODS

### *Drosophila* strains

The following fly strains were used in this study: Oregon-R, *wdp^KO^* (Takemura et al., 2020), *wdp-HA* (Takemura et al., 2020), *Sulf1^ΔP1^* (Kleinschmit et al., 2010), *dally^gem^* (Nakato et al., 1995; Tsuda et al., 1999), *trol-GFP* (Medioni and Noselli, 2005), *dpp^d-ho^* [Bloomington *Drosophila* Stock Center (BDSC) #308], *upd^os-s^* (BDSC #79), *hsp70-flp* (BDSC #8862), *ap-Gal4*, *hh-Gal4*, *mef2-Gal4* (BDSC #27390), *Bx-GAL4* (BDSC #8860), *UAS-GFP* (BDSC #1521), *nub-GAL4* (BDSC #25754), *Act5C-Gal4* (BDSC #3954), *Act5C>CD2>Gal4* flp-out cassette (Fujise et al., 2003), *UAS-wdp* (Takemura et al., 2020), *UAS-wdp RNAi* (TRiP.HM05118, BDSC #28907), *UAS-Chsy RNAi* [GD14159, Vienna *Drosophila* Resource Center (VDRC) #29084], *UAS-C4ST RNAi* (*UAS-CG31743.IR.Y*) (Yamamoto-Hino et al., 2015), *UAS-ttv RNAi* (GD1993, VDRC #4871) and *UAS-sfl RNAi* (HMS00543, BDSC #34601). The genotypes of fly strains used for the data shown in the figures are listed in Table S1.

Flies were raised on a standard cornmeal fly medium at 25°C unless otherwise indicated. For the Dpp signaling assay, flp-out clones overexpressing *wdp* were generated as previously described (Bowden et al., 2022; Struhl and Basler, 1993) in wing discs bearing an *Act5C>CD2>Gal4* transgene cassette, *hsp70-flp*, *UAS-GFP* and *UAS-wdp*. The FLP expression from *hsp70-flp* was induced by heat-shock treatment of larvae at 37°C for 30 min at 30–40 h after egg laying. For the Wg signaling assay, we overexpressed *wdp* with *ap-Gal4* during third larval instar stage by a temperature shift.

### Preparation of adult wings

The right wings from female flies were dehydrated in ethanol and subsequently with xylene (Fujise et al., 2001; Takemura et al., 2020). The specimens were mounted in Canada balsam (Benz Microscope, BB0020).

### Immunohistochemistry, immunoblot analysis and coimmunoprecipitation

Immunostaining of the wing discs and ovaries was performed as previously described (Hayashi et al., 2009, 2012; Takemura et al., 2020). The primary antibodies used were as follows: rat anti-HA 3F10 (1:200, Roche, 11867423001), rabbit anti-HA C29F4 (1:1000, Cell Signaling Technology, 3724), rabbit anti-pSmad3 (1:1000, Epitomics, 1880-1), guinea pig anti-Sens (1:1000, a gift from Hugo Bellen, Baylor College of Medicine, TX, USA), mouse anti-Dll (1:400, a gift from Dianne Duncan, Washington University in St. Louis, MO, USA), mouse anti-CS-A (1:100, Tokyo Chemical Industry, LY111), mouse anti-Fasciclin III 7G10 [1:50, Developmental Studies Hybridoma Bank (DSHB)] and rabbit anti-Vas (1:500, a gift from Satoru Kobayashi, University of Tsukuba, Tokyo, Japan). The secondary antibodies used were Alexa Fluor 488, 568 or 633 conjugated (1:500, Thermo FisherScientific). Extracellular labelling of Wg protein was performed as described previously (Kleinschmit et al., 2010; Strigini and Cohen, 2000) using the anti-Wg antibody (4D4, DSHB) at a 1:3 dilution. Images were obtained using a Zeiss 710 laser scanning confocal microscope.

For immunoblot analysis, protein samples were extracted from *Drosophila* adult whole body (for CS detection) or adult ovaries (for Wdp–HA detection) by SDS sample buffer. Mouse anti-CS A (1:1000, Tokyo Chemical Industry, LY111), rat anti-HA antibody (3F10) (1:2000, Roche, 11867423001), and mouse anti-α-tubulin (1:2000, Sigma-Aldrich, DM1A) were used as primary antibodies. Signals were detected using HRP-conjugated secondary antibodies (goat anti-mouse IgG Fc-HRP and goat anti-rat IgG-HRP obtained from SouthernBiotech, Birmingham, AL) and Pierce ECL Western Blotting Substrate (Thermo Fisher Scientific). For blot transparency, original immunoblots are given in Fig. S1.

For coimmunoprecipitation experiments, we generated a construct for a secreted form of Myc–Wdp (sec-Myc–Wdp) by deleting the transmembrane domain and the intracellular domain (A493–H719). *Drosophila* Dmel2 tissue culture cells were transfected with pMT-Wg (Kleinschmit et al., 2010) and/or pAW-sec-Myc–Wdp (this study). After incubation at 25°C for 72 h, 1 ml of each conditioned medium was incubated with anti-cMyc monoclonal antibody-agarose beads (Sigma-Aldrich) overnight at 4°C, washed, eluted with 6 M urea and analyzed by immunoblotting.

### Preparation and structural analysis of *Drosophila* GAGs and CSPGs

To isolate *Drosophila* GAGs, approximately 1.0 g of lyophilized adult flies was defatted with acetone and then extracted with 0.5% SDS, 0.1 M NaOH and 0.8% NaBH_4_ as previously described (Toyoda et al., 2000). The crude GAGs were applied to a HiPrep DEAE 16/10 column [16 mm internal diameter, 100 mm length; GE Healthcare (Uppsala, Sweden)] equilibrated with 25 mM phosphate buffer (pH 6.0) and elution was performed with a 0–1.5 M NaCl gradient in the same buffer at a flow rate of 1.0 ml/min.

To isolate *Drosophila* CSPGs, approximately 1.2 g of lyophilized adult flies was defatted with acetone. The samples were treated with 4 M guanidinium chloride, 0.05 M phosphate buffer, pH 6.0, and 1% Triton X-100, containing proteinase inhibitors (cOmplete™ ULTRA Tablets, Mini, EDTA-free, EASYpack obtained from Roche), for 2.5 h at room temperature with constant stirring. The extract was centrifuged at 20,000 ***g*** for 10 min to remove insoluble materials. The crude CSPG fractions were dialyzed into distilled water and then into 25 mM phosphate buffer, pH 6.0. The resulting solution was separated by anion-exchange chromatography using a Hi Trap DEAE FF (16 mm×50 mm) column (GE Healthcare) at a flow rate of 2 ml/min. The column was equilibrated with 25 mM phosphate buffer (pH 6.0), 0.5 M urea and 0.02 M NaCl, and eluted stepwise with increasing concentrations of NaCl at 0.26 M and 1.0 M. The eluents were monitored at 280 nm. The fractions (0.26 M and 1 M NaCl) were desalted and dissolved in 4 ml of water.

Disaccharide composition analysis was carried out as previously described (Dejima et al., 2013b; Kamimura et al., 2006; Kleinschmit et al., 2010; Nakato et al., 2019; Toyoda et al., 2000). Briefly, a 20 µl portion of the sample solution was incubated with 5 µl of 0.2 M Tris-acetate buffer (pH 8.0) and 10 µl of an aqueous solution containing chondroitinase ABC or ACII [1 mIU; chondroitinase ABC (EC 4.2.2.4) and chondroitinase ACII (EC 4.2.2.5) were obtained from Seikagaku, Tokyo, Japan] at 37°C overnight. The resulting disaccharide species were separated using reversed-phase ion-pair chromatography [Docosil C22 (4.6×150 mm; particle size, 5 μm) was obtained from Senshu Scientific, Tokyo, Japan]. The effluent was monitored fluorometrically for post-column detection of CS or HS disaccharides (Toyoda et al., 2000).

## Supplementary Material

10.1242/joces.260525_sup1 Supplementary information
