# Supplementary material for: Regulation of morphogen pathways by a Drosophila chondroitin sulfate proteoglycan Windpipe
Source: J Cell Sci. 2023 Apr 11;136(7):jcs260525. doi: 10.1242/jcs.260525 (PMC10113886; doi:10.1242/jcs.260525)
Supplement: Supplementary information [file joces-136-260525-s1.pdf]

Table S1. Genotypes of *Drosophila* strains used in each figure

Figure	Panel	Genotype
1	A-G	+/+
1	D-G	*wdp^{KO}/wdp^{KO}*
1	D	*Act5C-Gal4/UAS-$Chsy^{RNAi.GD14159}$*
1	D	*wdp-HA/+*
1	D	*Act5C-Gal4/UAS-$Chsy^{RNAi.GD14159}$; wdp-HA/+*
2	A, D	+/+
2	B	*UAS-$Chsy^{RNAi.GD14159}$/+; hh-Gal4 UAS-GFP/+*
2	C, D	*wdp^{KO}/wdp^{KO}*
2	E	*hh-Gal4 UAS-GFP/UAS-$wdp^{RNAi.HM05118}$*
2	F	*UAS-C4ST (UAS-CG31743.IR.Y)/+; hh-Gal4 UAS-GFP/+*
2	G	*trol-GFP/+; wdp-HA/+*
3	A, D	*Bx^{MS1096}-Gal4/+*
3	B, D	*Bx^{MS1096}-Gal4/+; UAS-wdp/+*
3	C, D	*Bx^{MS1096}-Gal4/UAS-C4ST (UAS-CG31743.IR.Y); UAS-wdp/+*
4	A, E, H	+/+
4	B-D	*nub-Gal4/+; UAS-wdp/+*
4	F-H	*hs-flp/+; Act5C>CD2>Gal4 UAS-GFP/+; UAS-wdp/+*
5	A, E, H	+/+
5	B-D, F	*nub-Gal4/+; UAS-wdp/+*
5	G, I, J	*ap-GAL4 UAS-GFP/+; UAS-wdp/+*
5	K, L	*hs-flp/+; Act5C>CD2>Gal4 UAS-GFP/+; UAS-wdp/+*
5	M	*hs-flp/+; Act5C>CD2>Gal4 UAS-GFP/+; UAS-3xMyc-$wdp^{\Delta GAG}$/+*
6	A, J, M	+/+
6	B	*wdp^{KO}/wdp^{KO}*
6	C	*$Sulf1^{\Delta P1}$/$Sulf1^{\Delta P1}$*
6	D-I, K, L, N, O	*wdp^{KO}/wdp^{KO}; $Sulf1^{\Delta P1}$/$Sulf1^{\Delta P1}$*
7	A, B, E, J, K	+/+
7	C, D, H, I, J, L	*wdp^{KO}/wdp^{KO}; $Sulf1^{\Delta P1}$/$Sulf1^{\Delta P1}$*
7	F	*wdp^{KO}/wdp^{KO}*
7	G	*$Sulf1^{\Delta P1}$/$Sulf1^{\Delta P1}$*
8	A, C, F	+/+
8	B, E, I	*wdp^{KO}/wdp^{KO}; $dally^{gem}$/$dally^{gem}$*
8	D, H	*$dally^{gem}$/$dally^{gem}$*
8	G	*wdp^{KO}/wdp^{KO}*
8	J	*dpp^{d-ho}/dpp^{d-ho}*
8	K	*upd^{os-s}/upd^{os-s}*
8	L	*Mef2-Gal4/UAS-ttv $RNAi^{GD1993}$*
8	M	*Mef2-Gal4/UAS-sfl $RNAi^{HMS00543}$*

Journal of Cell Science • Supplementary information

Table S2. Disaccharide analyses of CS from wild-type and *wdp* mutant adults.
Disaccharide composition of CS is shown for each respective genotype. The values are given as mol% of total disaccharides, and represent mean ± S.D. from three independent experiments. Graphical depiction of this result is shown in Fig. 1E and F.

	CS (unsaturated disaccharide, %)		Total amount
	**ΔDi-0S**	**ΔDi-4S**	**(ng/mg)**
**wild-type**	73.1±0.4	26.9±0.4	159.0±4.7
wdp	75.1±0.3	24.9±0.3	128.9±12.9

Table S3. Disaccharide analyses of HS from wild-type and *wdp* mutant adults.
Disaccharide composition of HS is shown for each respective genotype. The values are given as mol% of total disaccharides, and represent mean ± S.D. from three independent experiments. NAc, ΔUA-GlcNAc; NS, ΔUA-GlcNS; NAc6S, ΔUA-GlcNAc6S; NS6S, ΔUA-GlcNS6S; 2SNS, ΔUA2S-GlcNS; and 2SNS6S, ΔUA2S-GlcNS6S. A graph showing total amount of HS is shown in Fig. 1G.

	HS (unsaturated disaccharide, %)						Total amount
	**NAc**	**NS**	**NAc6S**	**NS6S**	**2SNS**	**2SNS6S**	**(ng/mg)**
**wild-type**	44.9±5.2	20.9±2.2	1.8±0.2	16.1±1.9	13.3±1.3	3.0±0.7	17.7±1.6
wdp	45.4±4.6	21.3±2.4	1.6±0.1	14.9±0.7	14.0±1.3	2.9±0.4	25.5±1.5

Journal of Cell Science • Supplementary information

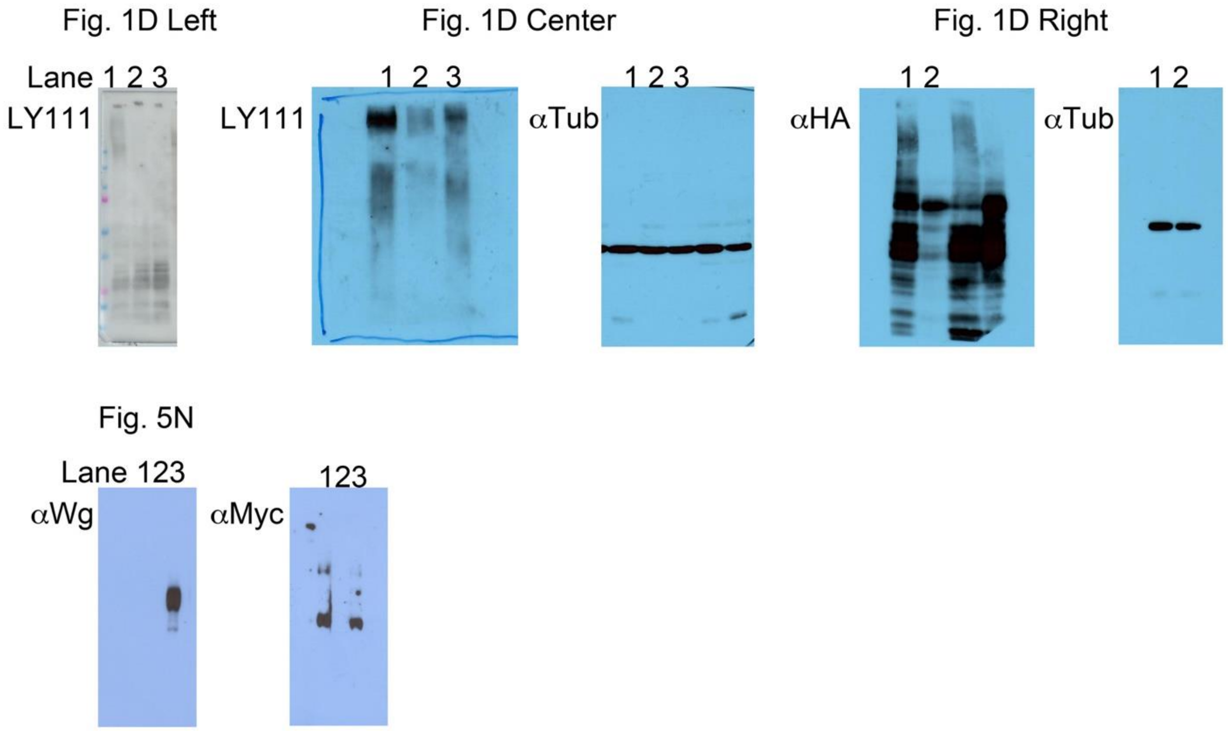

Fig. S1. Blot transparency.
Original Immunoblot data used for Fig. 1D and Fig. 5N are shown. Only the lanes with lane numbers were used in each panel. Antibodies used are indicated.
